# Miscellaneous Intelligence

**Published:** 1849-11

**Authors:** 


### ARTICLE IV.

### MISCELLANEOUS INTTLLIGENCE.

*Illinois General Hospital of the Lake.*---We are gratified to be able to announce the passage by the recent Extra Session of the State Legislature of Illinois, a bill chartering the above named benevolent Institution. The provisions of the charter are liberal, and it is expected that arrangements will be made to put it into operation at an early day.

*Chicago Marine Hospital.*---The foundation of this noble Institution has been laid, and the walls will go up early next spring.

*State Medical Convention.*---Just as our last form is going to press, too late for insertion, we have received a communication from Dr. E. A. Guilbert, Secretary of the Ottowa Medical Society, notifying us of a change of the time of meeting to the first Monday in May next. As this will be the day before the next meeting of the National Medical Association in Cincinnati, we hope the call has not yet been made general. A large number of the members of the profession who would take an active interest in the State Convention, would still prefer to attend the great National Meeting at Cincinnati. We would suggest to the Society the first Tuesday in June next as a good time for the State Convention, and promise to give the influence of our Journal to the enterprise.

*Indiana Central Medical College*.---This Institution is announced to go into operation on the first Monday of the present month, under the auspices of the following Faculty:---Obstetrics, &c., Geo. W. Mears, M. D.; Theory and Practice, L. Dunlap, M.D.; Anatomy, Jno. S. Bobbs, M.D.; Physiology and Pathology, R. Curran, M.D.; Surgery, A. H. Baker, M.D.; Materia Medica, &c., Jas. S. Harrison, M.D.; Chemistry, Ch. G. Downey, A.M.; Demonstrator of Anatomy, David Funkhouser, M.D.

*Medical College of Evansville*.---We notice this institution is advertised to go into operation on the first Monday in the present month, under the direction of the following faculty:---Anatomy, C. S. Weaver, M.D.; Chemistry, C. A. Foster, A.M.; Mat. Med., J. R. Wilcox, M. D.; Surgery, M. J. Bray, M.D.; Practice, L. S. Laycock, M.D.; Obstetrics, &c., Geo. B. Walker, M.D. Fees: Profs. Tickets, $10 00 each.

This is the third Medical School now in operation in Indiana, one being located at each extreme, and one in the centre of the State.

*Rock Island Medical School*.---The location of this School has been changed to Davenport, Iowa, and its style and designation altered to the "College of Physicians and Surgeons of the Upper Mississippi."

*Franklin Medical College* is announced to go into operation the present Autumn, at St. Louis.

